# Aflibercept as a Treatment for Secondary Central Serous Chorioretinopathy in a Patient With Myasthenia Gravis

**DOI:** 10.7759/cureus.31287

**Published:** 2022-11-09

**Authors:** Estefania Ramirez Marquez, Guillermo A Requejo Figueroa, Mariella Pappaterra-Rodriguez, Sofía C Ayala Rodríguez, Guillermo Puebla, Ileana Nieves, Armando L Oliver

**Affiliations:** 1 Ophthalmology, University of Puerto Rico School of Medicine, Medical Sciences Campus, San Juan, USA

**Keywords:** case report, serous retinal detachment, amblyopia, aflibercept, central serous chorioretinopathy

## Abstract

We report on a case of central serous chorioretinopathy (CSCR) secondary to chronic steroid use that showed sustained improvement when treated with an aflibercept intravitreal injection. A 44-year-old woman presented with decreased visual acuity of the left eye (OS). The patient had a recent history of myasthenia gravis and was being treated with systemic corticosteroids and immunosuppressants. At presentation, her visual acuity was 20/80 OS; an examination (using fluorescein angiography) of the left fundus revealed a serous retinal detachment of the posterior pole that extended to the mid-periphery and multiple areas of leakage, which findings were consistent with CSCR. The patient also had a history of unresolved strabismic amblyopia in her right eye. The patient’s CSCR was managed with one injection of intravitreal aflibercept (2 mg/0.05 mL). One month following treatment, her visual acuity improved to 20/20 OS, and the serous retinal detachment had resolved. Ten months following treatment, an examination revealed a sustained improvement, with a visual acuity of 20/20 OS. Concomitantly, the patient’s amblyopic eye revealed an improved visual acuity of 20/20. Our case suggests that some cases of secondary CSCR may respond to treatment with intravitreal aflibercept. This case also suggests that the CSCR imposed a unique form of occlusion therapy that helped improve the amblyopia of the contralateral eye in this adult patient.

## Introduction

Central serous chorioretinopathy (CSCR) is characterized by serous detachments of the neurosensory retina, most commonly involving the posterior pole [1-5]. Central serous chorioretinopathy has a multifactorial etiology; some risk factors include type-A personality, Cushing syndrome, pregnancy, hormonal contraceptives, and oral or inhaled corticosteroids [1,6]. Anatomical factors, such as pachychoroid or venous choroidal anastomoses, may also predispose an individual to this condition [7-9].

Multiple treatment options for managing CSCR include focal laser photocoagulation, photodynamic therapy, oral mifepristone administration, and anti-vascular endothelial growth factor (VEGF) pharmacotherapy [2,4-6,10,11]. However, it is of utmost importance to identify modifiable risk factors. For instance, when the etiology is suspected to be secondary to chronic steroid use, the tapering down or complete discontinuation of the inciting medication should be considered [2,4,6]. In some circumstances, the cessation of systemic steroids may be infeasible as such an action may exacerbate the systemic disease and possibly threaten the patient’s life. Laser or pharmacologic therapy may often be required to improve a given patient’s CSCR [6].

We hereby present the case of a woman with unilateral CSCR secondary to steroid therapy for systemic manifestations of myasthenia gravis and amblyopia of the contralateral eye and whose CSCR was managed with a single intravitreal aflibercept injection. This treatment resulted in the resolution of subretinal fluid and improved her visual acuity. This article was previously presented as a conference poster at the 2022 Annual Research and Education Forum on March 30, 2022.

## Case presentation

A 44-year-old female presented with a gradual worsening of visual acuity, micropsia, and metamorphopsia in the left eye (OS) for a duration of approximately two weeks. The patient had been diagnosed with myasthenia gravis, which presented with horizontal diplopia, bulbar symptoms, and extremity weakness four months earlier. Subsequently, she began treatment with pyridostigmine (60 mg daily), prednisone (40 mg daily), azathioprine (100 mg two times a day), and IV immunoglobulin. The patient had a history of unresolved strabismic amblyopia in the right eye (OD) since childhood, following the prior surgical correction of her strabismus. The patient received medroxyprogesterone acetate (150 mg/mL by injection) one month prior to presenting at our clinic. Previous attempts to taper the prednisone dose had exacerbated her myasthenia symptoms, which were stable upon her presentation at our clinic.

**Figure 1:**
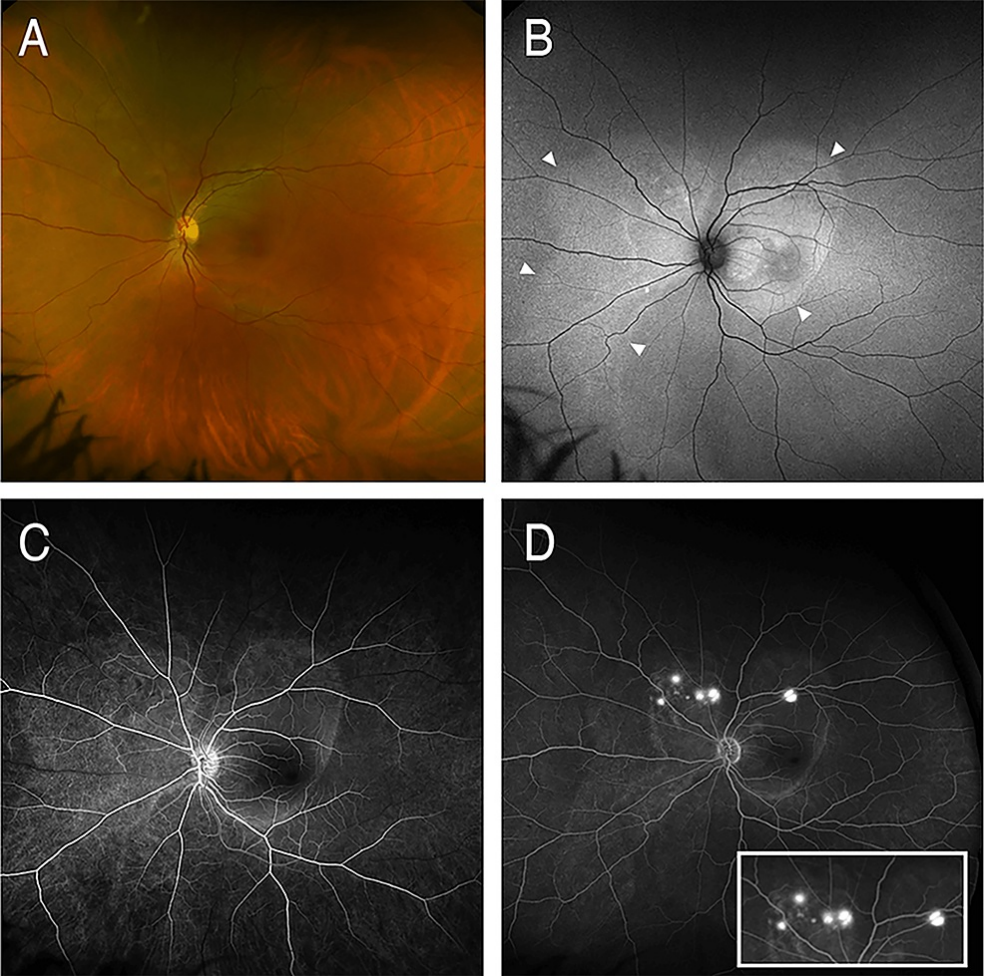
Ultra-widefield fundus imaging and optical coherence tomography of the left eye. Color photograph (A) reveals a serous retinal detachment of the posterior pole and the nasal mid-periphery, with corresponding hyperautofluorescence (arrowheads) (B). The early arteriovenous phase of the fundus fluorescein angiogram (FFA) (C) reveals a well-demarcated area of hyperfluorescence, corresponding with the serous retinal detachment. Late-phase (D) FFA reveals multiple clustered areas of multifocal leakage (inset).

**Figure 2:**
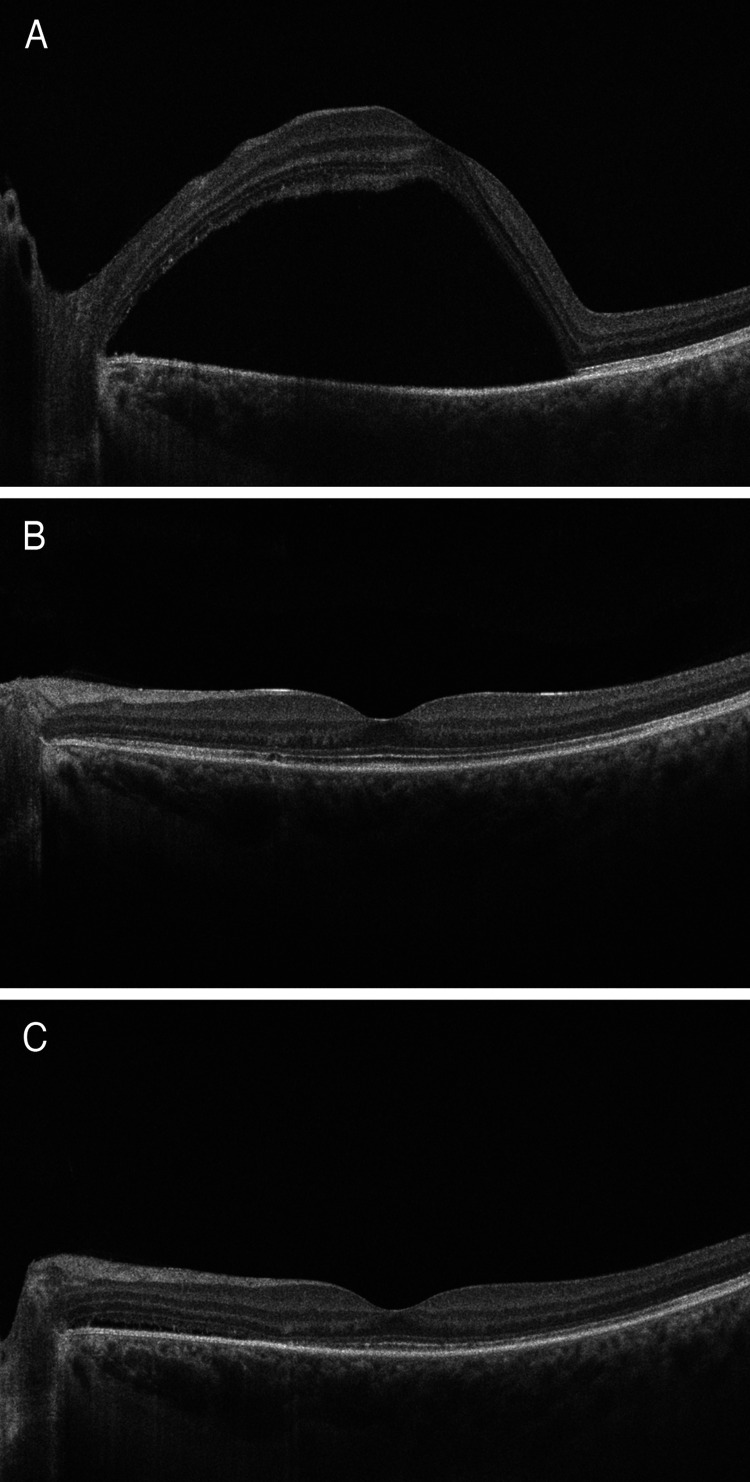
Spectral-domain optical coherence tomography of the left macula. The images at presentation revealed (A) a severe macular serous detachment. One month following therapy (B), a complete foveal reattachment was achieved, which persisted until the patient’s final visit (C) 10 months following the intravitreal aflibercept therapy.

A comprehensive ophthalmological exam revealed a best-corrected visual acuity of 20/70 in the OD and 20/80 in the OS with a manifest refraction of plano OD and +0.75 -0.25 x 160 OS. Intraocular pressures were 13 mmHg in both eyes. The patient was orthotropic with complete extraocular movements, and no detectable ocular paresis. A slit-lamp examination of the anterior segment was unremarkable in both eyes, without any evidence of inflammation. An examination of the left fundus revealed a serous retinal detachment of the posterior pole and the nasal mid-periphery, with corresponding areas of fundus autofluorescence (Figures 1A, 1B). Spectral-domain optical coherence tomography of the macula revealed a severe serous retinal detachment (Figure 2A). The fluorescein angiogram (FFA) revealed well-demarcated areas of early hyperfluorescence along with multiple foci of late-phase fluorescein leakage (Figures 1C, 1D). Indocyanine green angiography (ICG) was deferred due to the patient’s history of a severe shellfish allergy.

An assessment of CSCR secondary to systemic corticosteroid use was made. As the patient’s CSCR was severe and involved her non-amblyopic eye, multiple laser, and pharmacological options were discussed with the patient and considered for immediate therapy. The patient agreed to the off-label use of intravitreal aflibercept as therapy. The patient was prepped and draped sterilely, and 2 mg/0.05 cc of aflibercept was administered intravitreally OS via pars plana.

One month following treatment, the patient’s uncorrected visual acuity had significantly improved to20/40 OD and 20/20 OS, and there was foveal reattachment with a near total resolution of the patient’s serous detachment (Figure 2B). Eight months following her initial presentation, systemic corticosteroid therapy had been reduced to 20 mg, which dose she remained on until her last follow-up. On the patient’s most recent follow-up visit, ten months after the initial presentation, her visual acuity remained stable, denoting a persistent improvement of her amblyopia.** **As the fovea remained attached (Figure 2C), the patient's central vision acuity was not compromised by the subtle layer of extrafoveal subretinal fluid, we chose not to administer an additional dose of aflibercept.

## Discussion

Anti-VEGF medications alter vascular permeability, which is the rationale for their use in CSCR [10,11]. The function of anti-VEGF is to bind VEGF molecules and inhibit their biological function. By this mechanism, anti-VEGF has been proposed to lead to choroidal vasoconstriction, increased membrane thickness, and possibly reduced vascular fenestrations [5,10,11]. The inhibition of multiple members of the VEGF molecular family, including VEGF-B and placental growth factor (PIGF), may be associated with a greater ability to decrease the permeability and leakage of the choroidal membrane [5,10,11].

Aflibercept is an anti-VEGF compound that acts as a decoy receptor blocking the binding of the VEGF-B and PIGF to VEGF receptor [10]. Because the molecule of anti-VEGF medication aflibercept is intermediate-sized, 115 kDa compared to the 48 kDa of ranibizumab and the 148 kDa of bevacizumab, it moves more readily through the target tissues [10,11]. Aflibercept also has a longer intravitreal half-life than other anti-VEGF medications, allowing for a longer duration of clinical action, which reduces the need for repeated treatments at relatively short intervals [10,11]. These features, among others, have been hypothesized to contribute to this medication's increased efficacy and sustainable results in inhibiting pathological vascular remodeling [10,11].

Central serous chorioretinopathy may predispose patients to choroidal neovascularization and polypoidal choroidal vasculopathy (PCV) [8]. The latter involves an intricate branching of vessels and aneurysmal lesions that may be driven by the VEGF family [7-9]. In our particular case, pharmacotherapy of CSCR with aflibercept may have also targeted a pathological PCV component leading to the substantial reduction of our patient’s subretinal fluid and an increase in visual acuity. Although we could not obtain an ICG angiogram due to the patient’s known allergy to shellfish, her FFA’s particular multifocal leakage, which had a clustered pattern, led us to consider the possibility of an underlying PCV component. Certainly, CSCR may improve spontaneously in some cases [6]. However, in our patient, the sizeable serous detachment and the secondary etiology, due to an unmodifiable risk factor at the time, render this possibility rather unlikely.

During the first month of her CSCR in the OS, the strabismic amblyopia of the OD improved. Amblyopia has traditionally been considered irreversible after the critical period for neuronal plasticity has passed when a child surpasses approximately 12 years of age [12]. However, many recent studies have shown that patients beyond the critical period may nevertheless experience improvements in their visual acuity with occlusion therapy [13,14]. Alternatively, penalty therapy for amblyopia may be achieved by reducing an eye's accommodative capacity through pharmacological treatments [12,15,16]. It is possible that the hyperopic shift caused by the severe CSCR significantly reduced the residual accommodative capacity in this quadragenarian patient, serving as a form of penalty therapy. The patient’s improvement in strabismic amblyopia supports the novel notion that some cases may be reversible, even in adulthood [13,14].

## Conclusions

Our case suggests that intravitreal aflibercept therapy may have a role in managing some secondary central serous chorioretinopathy cases. Such treatment may be beneficial when concomitant systemic disease does not allow the tapering of systemic steroids without risking the patient’s overall well-being. It also suggests that penalty therapy, resulting from a reduced accommodative amplitude secondary to a hyperopic caused by a sizeable serous macular detachment in the contralateral eye, may serve as treatment and result in the improvement of some cases of long-standing amblyopia in adults.
